# Association between family factors and 24-h movement behaviors of adolescents—a cross-sectional study of Chinese high school students

**DOI:** 10.3389/fpubh.2025.1564423

**Published:** 2025-05-19

**Authors:** Huiying Fan, Guoqiang Fan, Jie Meng

**Affiliations:** ^1^School of Physical Education, Shanghai Normal University, Shanghai, China; ^2^Department of Sports, Shanghai University of Traditional Chinese Medicine, Shanghai, China; ^3^School of Sports and Health, Linyi University, Linyi, China

**Keywords:** family factor, 24-h movement behavior, adolescent, cross-sectional study, Chinese high school student

## Abstract

**Background:**

Families have a significant effect on the patterns of physical activity exhibited by adolescents, and they influence the movement behaviors of adolescents through both direct and indirect roles. The aim of this study was to examine the association between meeting the 24-h movement guidelines and family factors.

**Methods:**

A total of 1,250 students (mean age16.1 ± 0.9 years; 48.2% were boys.) were selected from the 10th, 11th, and 12th grades in four urban and four suburban districts in Shanghai, China. The study used generalized linear models to assess the association between adherence to the 24-h movement behaviors and family factors, while adjusting gender, ethnicity, age, weight status, live in school. For statistical significance, *p* value <0.05 was used.

**Results:**

Adolescents in nuclear families (OR = 1.273, CI = 0.031–0.451, *p* = 0.024), with no siblings (OR = 1.108, CI = 0.033–0.173, *p* = 0.004), middle (OR = 1.106, CI = 0.018–0.183, *p* = 0.017), and high SES (OR = 1.160, CI = 0.055–0.242, *p* = 0.002) were more likely to meet the 24-h movement behavior guidelines. For boys, middle (OR = 1.221, CI = 0.071–0.329, *p* = 0.002) and high (OR = 1.289, CI = 0.107–0.399, *p* = 0.001) SES were more likely to meet the 24-h movement behavior guidelines. Girls with nuclear families (OR = 1.488, CI = 0.121–0.674, *p* = 0.005) and no siblings (OR = 1.125, CI = 0.029–0.207, *p* = 0.009) were more likely to meet the 24-h movement behavior guidelines.

**Conclusion:**

This study demonstrated the role of family structure, having siblings, and SES in predicting adolescent 24-h movement behavior. The findings of the research assistance in providing information family factors for future interventions aimed at establishing adolescent 24-h movement behaviors. The mechanisms by which these family factors influence 24-h movement behavior should be explored in future, and the specific pathways by which family factors influence adolescent 24-h movement behavior should be clarified, in order to provide a more targeted theoretical basis for interventions.

## 1 Introduction

Adolescence represents a pivotal stage in human development for an individual’s physical development and cognitive development (1, 2), as well as for the formation of healthy lifestyle habits and behaviors that can continue into adulthood (3). Elevated levels of physical inactivity and sedentary behaviors are a primary factor of death globally, leading to many health problems for instance obesity, cardiovascular disease and mental illness (4). There exists substantial that patterns of 24-h movement behaviors, including physical activity, sedentary behavior and sleep duration, affect biological processes over the course of a 24-h duration and may influence health-related outcomes in adolescents (5).

Based on an comprehension of the health consequences of 24-h movement behaviors, Canada developed and presented the first 24-h movement guidelines for children and adolescents (6). Since then, the World Health Organization, Australia, New Zealand, South Africa and have implemented 24-h movement guidelines for children and adolescents across various age groups (7–9). Unlike previous guidelines that concentrated on moderate to virgate intensity physical activity (MVPA), the 24-h movement guidelines include recommendations for screen time (ST) and sleep. Unfortunately, less than 10% of adolescents globally fail to adhere to the suggested levels outlined in 24-h movement guidelines, and a significant number of adolescents fail to adhere to the established guidelines for physical activity (10).

Families have a significant influence on the movement behavior of adolescents, with parents influencing their children’s movement behavior strategies through both direct and indirect roles. The study of parental perceptions of child movement behavior investigated that and family environment have a strong influence on the engagement of children and adolescents in physical activity (11). A large study of parent–child movement behavior based on objective measurement data reported that 10–16% of MVPA and between 41 and 46% of sedentary behaviors were observed to occur concurrently, with the predominant setting for these activities being within the home environment (11). Family-centered interventions are more effective in promoting health impacts and movement behavior in adolescents, and sustained alterations in the physical activity levels of adolescent over an extended period are difficult to achieve without family involvement (12). Evidence shows that parental influence on adolescents’ physical activity, either positively or negatively, may depend on the structure of the family in which the child grows up. Adolescents in reconstituted families or single-parent are associated with less physical activity, more screen time (13) and poor sleep (14). In addition, family economic status has a significant impact on adolescents’ movement behavior, with adolescents from well-off families experiencing better physical activity, fewer screen time (15), and better-quality sleep (16).

Given the significance of the family involvement in the physical activity, screen time, and sleep of adolescents. Canada released the formulation of a consensus statement regarding the influence of family dynamics on the physical activity, sedentary behavior, and sleep patterns of children and adolescents in 2020 (17). The study highlights the significance of family for children’s movement behaviors as well as the significance of using the whole family system as a source of influence and promoting healthy children and adolescents’ movement behavior. Taken together, these reasons make parental 24-h movement behaviors important to the study of adolescent 24-h movement behavior and the development of interventions (18). But as we know, the association between combinations of sleep time, screen time, and MVPA and family remains to be explored. The impact of familial factors on the three types of movement behaviors is complex and intersectional, with most of the sleep being accomplished in the home, but the physical activities and screen time occurring both within the home and in external environments. The influence of family factors on the movement behaviors cannot be viewed in isolation.

Several studies have demonstrated gender differences in the association between family factors and 24-h movement behavior. A study by Cheng et al. showed that family financial support had a more significant effect on boys’ physical activity (19). This may be due to the fact that boys tend to participate in higher levels of physical activity than girls, and higher levels of physical activity require higher levels of financial support. In addition, girls are more sensitive to family structure, and family conflict or discord affects girls more than boys (20). These previous findings indicate that there may be gender differences between family factors and adolescents’ 24-h movement behaviors, which inspired us to explore this aspect in the present study.

24-hmovement behaviors are also an important way to influence the healthy development of Chinese adolescents. Lack of physical activity, prolonged screen exposure, and insufficient sleep are also commonly occurring among the youth population in China, specifically children and adolescents. Research shows that merely 5% of children and adolescents in China who are enrolled in grades 4 through 12 meet the 24-hour physical activity guideline (in a minimum of 60 min of moderate-to-vigorous physical activity daily, limit screen time to no more than 2 h per day, and 9 to 11 h and 8 to 10 h of sleep per night for 6–13 and 14–17 year olds, respectively) (21). Some studies have shown that family may have a significant effect on physical activity (22), sedentary time (23), and sleep (24) among Chinese children and adolescents. Unfortunately, no studies have examined the relationship between family factors and 24-h behavior in Chinese adolescents. Consideration of motor behaviors throughout the 24-h period contributes to adolescent cognition, growth and development, and mental health related to the close relationship between family and adolescent healthy development. Clarifying the association between family and 24-h movement behaviors will facilitate more evidence-based support for health interventions covering the whole day and the whole field for adolescents. Therefore, the aim of this study was to investigate the association between meeting the 24-h movement guidelines for session combinations of physical activity (≥60 min per day), screen time (≤2 h/day), and sleep duration (8–10 h per day for 14–17-year-olds) session combinations and adolescent family factors.

## 2 Methods

### 2.1 Participants and procedures

The participants involved this research were high school students in Shanghai, China. We randomly selected four urban and four suburban districts in Shanghai (urban: Jing’an, Yangpu, Huangpu, and Xuhui districts, and suburban: Pudong, Qingpu, Minhang, and Fengxian districts), one high school in each district was then randomly selected and contacted. After obtaining consent from the schools, two classes in each grade level were randomly invited to participate in the study.

The sample size estimation was performed utilizing G*Power software analysis complemented by empirical guidelines. First, *a priori* power analysis was conducted using G*Power 3.1 with the following parameters: medium effect size (Cohen’s *f* = 0.15), significance level (*α*) of 0.05, and statistical power (1-*β*) of 0.8. The results indicated that a minimum of 65 samples would be required to adequately detect correlations between variables. During the actual data collection process, the survey was administered to 1,322 adolescents in order to improve the statistical efficacy and external validity of the study. The validity of the data was judged according to whether the information in the questionnaire was filled out completely and whether the questionnaire was filled out in accordance with the norms of common sense (a questionnaire is considered invalid if the same option is chosen for 10 or more consecutive questions, and an invalid questionnaire is considered invalid if the physical activity, screen time, and sleep time are out of the normal range). If the questionnaire has missing values, the entire questionnaire is treated as invalid. Finally, the questionnaires of 1,250 people were considered valid. This sample size far exceeded the minimum requirements, and this large-scale sample not only strengthened the reliability of the analyses of the relationships between the study variables, but also increased the stability, generalizability and representativeness of the findings in the population.

The aims and procedures of the survey were communicated to the heads of physical education of the selected schools prior to the survey. The physical education teacher and the researcher then worked together to introduce the program to the students and ask for their wishes before the start of the survey, and students who did not want to take part were permitted to leave. An informational document outlining the study was accessible to all students during the testing period. Before the survey, students were informed that participation in the survey was voluntary, allowing them the option to either engage or decline involvement. The questionnaire was administered anonymously to the students. The Ethics Review Board of Shanghai Normal University gave us permission to conduct this study with approval number 2024080.

### 2.2 Variables and measurements

#### 2.2.1 24-h movement guidelines

Physical activity: Physical activity was assessed utilizing the International physical activity questionnaire-short form (IPAQ-SF). The questionnaire measures the physical activity of the respondents over the past week through nine questions. The short form tracks activity across four different intensity levels: (1) vigorous intensity physical activity (VPA) such as aerobic exercise, (2) moderate intensity physical activity (MPA), (3) walking, and (4) sitting. The scale has been validated internationally (25) and in the Chinese population (26). In this study, based on the participants’ answers, the PA requirement of the 24-h movement guideline was considered to be fulfilled when the count of days of MVPA in the previous week was equal to 7 days, and at the same time, the duration of MVPA was equal to or more than 60 min per day (not met guideline coded as 1, met coded as 2).

Screen time: Screen time measured using Health Behavior in School-aged Children (HBSC) questions (27). The questionnaire has been validated for its reliability and validity and has been used in studies of screen time among high school adolescents (28–30). Participants were asked 3 questions about daily screen-based sedentary patterns during free time (weekdays and weekends). Specifically, this included; (1) the duration of engagement with television, video content, and screen-based entertainment per day., (2) the duration of engagement in gaming activities on a computer, gaming console, or tablet/smartphone per day, and (3) the duration of engagement with electronic devices, including smartphones, computers, tablets or for purposes other than their primary functions was assessed per day. The response options for each inquiry adhered to a consistent format (no time at all, approximately 30 min, 1 h, 2 h, 3 h, 4 h, 5 h, 6 h, and seven or more hours per day). Participants with less than or equal to 2 h of screen time per day were regarded to have met the 24-h movement guideline, while those with more than 2 h were regarded have not fulfilled the 24-h movement guideline (not met guideline coded as 1, met coded as 2).

Sleep time: Record the average number of hours of sleep per night by asking: “In the past month, what was your average total hours of sleep per night?.” Based on the sleep recommendations for a specific age group (8–10 h for 14–17 year olds), determine if the sleep recommendations are being met (not met guideline coded as 1, met coded as 2).

Finally, the number of guidelines met by each respondent was calculated (the number of guidelines met ranged from 0 to 3) based on whether physical activity (MVPA≥60 min per day), screen time (≤2 h/day), and sleep duration (8–10 h per day for 14–17-year-olds) were met.

### 2.3 Family factor

Family socioeconomic status (SES): SES was calculated based on parental occupation, parental educational attainment, and monthly household income. Parental occupation was used as a standardized scale according to the Occupation Scale for Urban Residents in China, which was founded on the Occupational Classification proposed by Duncan (31) in combination with the Chinese situation, and has frequently been utilized in social science studies in China (32). Parents’ academic education was evaluated based on the years of education completed. 1–7 points according to no education, primary school, junior high school, secondary school, senior high school, university, postgraduate and above. Monthly household income was divided and assigned values according to less than 2,000 yuan per month (2 points), 2001–5,000 yuan per month(5 points), 5,001–8,000 yuan per month(8 points), and more than 8,000 yuan per month(10 points) (33). The standardized z-values of the above indicators were subjected to principal component analysis to obtain factor loadings for each variable. Family SES = (β1 × Z education + β2 × Z occupation + β3 × Z possession) / εf, where β1–3 are the factor loadings and εf is the eigenvalue for the first factor (34). The factor scores were equally divided into three categories according to the lowest to the highest: low, middle, and high (coded as 1–3) (35).

Having sibling: The participants were asked if they had siblings, and participants answered yes (coded as 1) or no (coded as 0).

Family structure: According to the type of family participants had, they were categorized into three groups: nuclear families (living with parents, coded as 1), single-parent families (living with the father or mother, coded as 2), and living with other relatives (living without parents, coded as 3).

### 2.4 Covariates

Sociodemographic variables were self-reported by the investigators and included age group, grade group, gender (boy/girl), body mass index (BMI), and ethnicity (Han or minority). Referring to the Chinese Classification Standards for Body Mass Index Values for Screening of Overweight and Obesity in School-Aged Children and Adolescents (36) (which classifies the BMI of children and adolescents of different ages and genders), they were categorized as no overweight, obese and overweight.

### 2.5 Statistical analysis

Generalized linear models to estimate the association between compliance with the 24-h movement behavior guideline and family factors, ordered logistic models were selected. The number of 24-h movement behavior guidelines met was used as the dependent variable, and family factors (family structure, having sibling, and SES) were used as the independent variables, while adjusting for gender, age, BMI, ethnicity, and whether or not they lived in the school as control variables. In order to examine whether there were differences between boys and girls in the association between 24-h movement behavior and family factors, we stratified the data by gender. Statistical analysis was conducted utilizing SPSS 24.0. Statistical significance was determined if the two-sided *p* value was < 0.05.

## 3 Results

Table 1 shows the characteristics of the study sample, which included 1,250 participants. The average age of the participants was 16.1 ± 0.9 years and 48.2% were boys. The highest number of participants was found in the 10^th^ grade followed by the 11^th^ grade and finally the12^th^ grade. The sample did not vary much between boys and girls, with 48.2% of boys and 51.8% of girls. The racial composition was predominantly Han at 97.0%. Overweight adolescents accounted for 13.0% and obesity for 6.7%. In terms of family factors, 82.4% of the samples were nuclear families, 15.0% of the samples were single-parent families (living with father or mother), and 2.6% of the samples were living with other relatives. Nearly half of the participants had siblings(48.3%), while half did not(51.7%). 10.2% of the participants slept 8 h a day, 74.7% of the participants had limit screen time to a limit of 2 h per day, and 8.6% of the participants were able to do a minimum duration of 60 min of physical activity a day. In addition, 20.6% of the participants failed to adhere to the 24-h movement behavior guidelines, 67.3% met only 1 guideline, 10.1% met 2 guidelines, and only 2.0% met 3 guidelines.

**Table 1:** Sample characteristics of this study (*N* = 1,250).

Sample characteristic	M ± SD	
Age	16.1 ± 0.9	
	n	%
Grade		
10th	580	46.4%
11th	357	28.6%
12th	313	25.0%
Sex		
Boy	602	48.2%
Girl	648	51.8%
Ethnicity		
Han	1,213	97.0%
Minority	37	3.0%
Weight status		
Non-overweight or obesity	1,003	80.2%
Overweight	163	13.0%
Obesity	84	6.7%
Live in school		
Yes	585	46.8%
No	665	53.2%
Family structure		
Nuclear families	1,030	82.4%
Single-parent family	188	15.0%
Living with other relatives	32	2.6%
Have sibling		
Yes	604	48.3%
No	646	51.7%
Family socioeconomic status		
Low	416	33.3%
Middle	417	33.4%
High	417	33.4%
Got 8 or more hours of sleep		
Not meet	1,123	89.8%
Meet	127	10.2%
Spent 2 or more hours/day on screen time		
Not meet	316	25.3%
Meet	934	74.7%
At least 60 min/day physical activity for all 7 days		
Not meet	1,123	89.8%
Meet	107	10.2%
Number of guidelines met		
0	258	20.6%
1	841	67.3%
2	126	10.1%
3	25	2.0%

PA, physical activity; SB, screen time; SLP, sleep.

Table 2 explores the association between compliance with the 24-h movement behavior guidelines and family factors. With respect to different family structures, adolescents in the nuclear families were more likely to meet the 24-h movement behavior guidelines compared to living with other relatives (OR = 1.273, CI = 0.031–0.451, *p* = 0.024). Compared to adolescents with siblings, the ratio of adolescents without siblings increased with the number of guidelines followed, suggesting that adolescents without siblings were more likely to meet the 24-h movement behavior guidelines (OR = 1.108, CI = 0.033–0.1 73, *p* = 0.004). For the different SES aspects, adolescents with middle SES (OR = 1.106, CI = 0.018–0.183, *p* = 0.017) and high SES (OR = 1.160, CI = 0.055–0.242, *p* = 0.002) had a ratio greater than low SES.

**Table 2:** Association between adherence to the 24-h movement guidelines and family structure in overall sample.

Family factor	OR	95%CI		*p*
Family structure (reference group = Living with other relatives)				
Nuclear families	1.273	0.031	0.451	0.024
Single-parent family	1.039	−0.184	0.262	0.732
Have sibling (reference group = yes)				
No	1.108	0.033	0.173	0.004
Family socioeconomic status *(reference group = low)*				
High	1.160	0.055	0.242	0.002
Middle	1.106	0.018	0.183	0.017

OR, odd ratio; CI, confidence interval. All models controlled for gender, ethnicity, age, weight status, live in school.

The sample is stratified by gender in Table 3. The presence of SES were significant factors for boys, with family structure and the presence of siblings having no significant effect. For boys, middle SES (OR = 1.221, CI = 0.071–0.329, p = 0.002) and high SES (OR = 1.289, CI = 0.107–0.399, *p* = 0.001) were more likely to meet the 24-h movement behavior guidelines. For girls, family structure and having siblings have a significant predictor, with no significant effect of SES. Girls with nuclear families (OR = 1.488, CI = 0.121–0.674, *p* = 0.005) and no siblings (OR = 1.125, CI = 0.029–0.207, *p* = 0.009) were more likely to meet the 24-h movement behavior guidelines.

**Table 3:** Association between adherence to the 24-h movement guidelines and family factors in sample by sex.

Family factor	OR	95%CI		*p*
Boy				
Family structure (reference group = Living with other relatives)				
Nuclear families	1.112	−0.207	0.418	0.508
Single-parent family	0.954	−0.383	0.288	0.782
Have sibling (reference group = yes)				
No	1.102	−0.012	0.204	0.081
Family socioeconomic status (reference group = low)				
High	1.289	0.107	0.399	0.001
Middle	1.221	0.071	0.329	0.002
Girl				
Family structure (reference group = Living with other relatives)				
Nuclear families	1.488	0.121	0.674	0.005
Single-parent family	1.140	−0.160	0.423	0.377
Have sibling (reference group = yes)				
No	1.125	0.029	0.207	0.009
Family socioeconomic status (reference group = low)				
High	1.044	−0.075	0.161	0.473
Middle	1.011	−0.093	0.116	0.833

OR, odd ratio; CI, confidence interval. All models controlled for gender, ethnicity, age, weight status, live in school.

## 4 Discussion

This study based on a population-based sample of Chinese high school students examined the association between adolescents’ family factors and 24-h movement behavior. Findings indicated that adolescents with no siblings, nuclear families, and higher SES have a higher likelihood of adhering to the 24-h movement behavior guideline recommendations. This finding aligns with existing literature. A scoping review study by Rhodes et al. research indicates that boy with two or more siblings engage in a greater amount of unstructured outdoor play during non-school hours than boys with one or fewer siblings; girls from two-parent households engaged in a greater number of organized sports activities., classes, and clubs than girls in single-parent families; and compared to single-parent families, two-parent families boys and girls in two-parent families engage in less television viewing; and, boys in two-parent families get more sleep than boys in single-parent families (17). A cross-sectional study of 24 countries in the European region showed significant differences in physical activity, sedentary behavior and sleep patterns among children and adolescent from varying socio-economic backgrounds (37).

The findings of this research indicate that adolescents in nuclear families exhibited a higher likelihood of meeting the 24-h recommendation amount compared to single-parent families and families living with other relatives. This finding aligns with another research. Langøy’s study showed that young people living with a single parent were inversely related to 60-min MVPA days/week and positively associated with total screen time hours/week. Young individuals raised by a single parent were less inclined to engage in physical activities. Whereas living in a reorganized family was inversely related to the 60-min MVPA days/week and positively associated with the hours of total screen time/week (13). Adolescents in single-parent and stepparent families more likely to experience reduced sleep duration during weekdays, longer sleep onset latency, longer waking time after onset of sleep, excessive sleeping, and difficulty falling asleep compared to their peers in nuclear families (14). Studies have found that children and adolescent in single-parent families receive reduced parental support resulting from time constraints, transportation, and supplementary parental obligations, which creates a home environment that encourages sedentary activity and a deficiency in sleep and physical activity supervision (38). While there are also studies that show that family structure does not have a significant effect on adolescents’ activity behaviors, such as McMilian’s study showing that young people from nontraditional families do spend slightly more time on screens per week, these differences are subtle (39). Sallis et al. concluded that the status of being a single parent has an inconclusive link with children’s PA (40). Chinese families are influenced by Confucianism and believe that the family constitutes the fundamental and essential unit of society (41). Many Chinese families believe that the common interests of the family are important, and traditional culture teaches family members how they should interact and how to maintain the common interests of the family. In the Chinese family structure, family members are very close to each other (42). Therefore, a closer family structure may allow adolescents in nuclear families to receive more family support and be more likely to achieve recommended amount levels of physical activity, screen time, and sleep.

This survey showed that adolescents without siblings had higher possibility to meet the 24-h movement behavior recommendations. Research findings on the number of children in the home on health behaviors are inconsistent. Gusrtafson’s study showed that boys without siblings watched more hours of television per day than boys with siblings. There were notable disparities in the duration of time spent by boys who have one sibling, or three or more siblings spent watching television. Girls with siblings spend more time per day on PA than only children (43). However, Ruseski’s research found that having infants or school-age adolescents in the home and caring for them reduces parental sport participation. Thus, the absence of children was positively associated with PA among family members. As the size of a family increases with the addition of children, the amount of time required for childcare also increases (44).Edwards’s research shows that the effects of activity behavior are more pronounced in informal and spontaneous physical activity, and that siblings exert a direct and measurable impact on what children watch (45). In single-parent families, boys with older male siblings and girls who grew up with siblings (regardless of gender) have higher levels of PA (40). This differential outcome may be related to cultural differences in families in different regions. China implemented the one-child policy in the last century, and the life and education of traditional Chinese families generally centered around one child. As the number of children increases, family supervision and management of adolescents’ activity behaviors are fragmented, and thus adolescents with siblings are less inclined to attain the 24-h movement behavior guideline recommendations.

Our research has found that adolescents from families with higher SES have a higher possibility to meet the recommended 24-h of movement behaviors than those from families with low SES. Data from the 2017/2018 Health Behavior in School-Aged Children show that adolescents from less affluent families participate in lower physical activity (27). A study including 24 countries in the WHO European Region showed that children and adolescent hailing from higher socioeconomic backgrounds exhibited a greater propensity to utilize motorized modes of transportation, and those from low socioeconomic status exhibited a lower propensity to engage in sports clubs and a higher likelihood of watching television for durations exceeding 2 h per day (37). Generally, families from varying socioeconomic backgrounds provide distinct forms of support to their children. Families from higher SES typically possess greater financial means to promote their children’s engagement in physical activity. As a result, parents of high SES are more inclined to promote the development of their children to be active in sports clubs, purchase high quality bedding, and other ways to safeguard their adolescents’ 24-h activity behaviors. Due to economic barriers, children of parents of lower SES may face limitations in their ability to engage in various extracurricular activities and are therefore more likely to opt for alternative solutions, including complimentary athletic programs in educational institutions and unstructured physical activities in communal areas, such as parks. It is notable, however, that there are other studies that show non-significant associations between SES and physical activity, screen time and sleep, such as 42% of studies that show the opposite or no association between SES and PA in adolescents (46). Potential explanations for these inconsistent findings include (a) heterogeneity in SES indicators, (b) mostly self-reported subjective measures of movement behavior, and (c) inconsistent measures of 24-h activity behavior (frequency vs. duration) (d) variability across countries. Considering the interrelated nature of the behaviors under investigation, subsequent research should prioritize the examination of patterns and clustering of adolescent movement behaviors, as well as their association with SES.

This study found gender differences in the association between family and 24-h movement behavior. To the best of our knowledge, no studies have examined these gender differences, but studies of single behaviors of 24-h movement behavior have presented similar gender differences. Cheng et al.’s study showed that financial support had a more pronounced effect on boys’ physical activity (19). This may be because boys tend to engage in higher levels of physical activity than girls, and higher levels of physical activity require higher levels of financial support to back it up (47). In addition, girls feel more sensitive to the structure of the family, and family conflict or discord affects girls more than boys (20). At the same time, the traditional Chinese cultural background may also contribute to gender differences. Traditional Chinese education expects boys to be more independent and autonomous, while girls expect their families to create a stable and caring environment for them (48). Therefore, even if there is a change in family structure, boys are more inclined to be independent of the family and mold themselves in external links such as school and community.

## 5 Strengths and limitations

Only a few studies have focused on the importance of the family’s association to 24-h movement behavior. Also, considering the diversity of family support in different country regions, this study is the first exploration of family factors and 24-h movement behavior in China. The results of this study show that the association between family structure, having siblings and socioeconomic status and adolescent 24-h movement behavior may be more complex in the Chinese cultural context. This complexity has not been fully explored in previous international studies, which have mostly focused on western cultures. In addition, the standardized methods for data collection and processing allowed comparative analyses between nations and increased the generalizability of our results. For future research, the mechanisms by which these family factors influence 24-h movement behavior should be explored to understand the specific mechanisms and pathways by which family factors influence adolescents’ behavior. For example, interviews with adolescents and their parents could provide informative data on how family factors influence 24-h movement behavior.

However, there are some limitations of this study. First, although the study controlled for gender, ethnicity, age, weight status, live in school, it may not have fully covered factors that influence all possible effects. Second, this study was a cross-sectional design, which limits the ability of this study to draw causal conclusions. Third, this study was based on self-reports, which may have some reporting bias, and adolescents may have underestimated their screen time and overestimated their physical activity and sleep. Fourth, the data were obtained only from Shanghai, China, which has unique characteristics such as regional culture, level of economic development, which may make it difficult to directly generalize the findings to other parts of China, and even more difficult to represent different countries and regions around the world. Fifth, there may be a potential aggregation effect in this study. Students in the same class or school may be subject to the same school environment, educational philosophy, and peer influences, which may lead to a certain degree of aggregation in the 24-h movement behavior and thus affect the accuracy of the final results.

## 6 Conclusion

This research demonstrated the role of family structure, having siblings, and SES in predicting adolescent 24-h movement behavior. The findings of the research contribute to the understanding of familial influences for future interventions aimed at establishing adolescent 24-h movement behavior. The mechanisms by which these family factors influence 24-h movement behavior should be explored in future, and the specific pathways by which family factors influence adolescent 24-h movement behavior should be clarified, in order to provide a more targeted theoretical basis for interventions.

## Data Availability

The raw data supporting the conclusions of this article will be made available by the authors, without undue reservation.
